# Widespread microglial activation in multiple system atrophy

**DOI:** 10.1002/mds.27620

**Published:** 2019-02-06

**Authors:** Dorothee Kübler, Tobias Wächter, Nicole Cabanel, Zhangjie Su, Federico E. Turkheimer, Richard Dodel, David J. Brooks, Wolfgang H. Oertel, Alexander Gerhard

**Affiliations:** ^1^ Movement Disorders Section, Department of Neurology, Charité‐Universitätsmedizin Berlin, corporate member of Freie Universität Berlin Humboldt‐Universität zu Berlin and Berlin Institute of Health Berlin Germany; ^2^ Hertie‐Institute for Clinical Brain Research Department of Neurodegenerative Diseases Tübingen Germany; ^3^ Department of Neurology Rehabilitation Centre Bad Gögging Passauer Wolf Bad Gögging Germany; ^4^ Vitos Clinical Centre for Psychiatry and Psychotherapy Giessen‐Marburg Germany; ^5^ Department of Neurosurgery Salford Royal NHS Foundation Trust Salford UK; ^6^ Department of Neuroimaging, Institute of Psychiatry, Psychology and Neuroscience King's College London London UK; ^7^ Chair of Geriatrics, University Hospital Essen Center for Geriatric Medicine Haus Berge Essen Germany; ^8^ Department of Nuclear Medicine and PET‐Centre Institute of Clinical Medicine, Aarhus University Aarhus C Denmark; ^9^ Institute of Neuroscience Newcastle University Newcastle Upon Tyne UK; ^10^ Department of Neurology Philipps‐Universität Marburg Marburg Germany; ^11^ Institute for Neurogenomics Helmholtz Center for Health and Environment München Germany; ^12^ Departments of Nulcear Medicine and Geriatric Medicine University Hospital Essen Germany; ^13^ Wolfson Molecular Imaging Centre University of Manchester Manchester UK

**Keywords:** microglia, multiple system atrophy, neuroinflammation, PET, PK11195

## Abstract

**Background:**

The pattern and role of microglial activation in multiple system atrophy is largely unclear. The objective of this study was to use [^11^C]*(R)*‐PK11195 PET to determine the extent and correlation of activated microglia with clinical parameters in MSA patients.

**Methods:**

Fourteen patients with the parkinsonian phenotype of MSA (MSA‐P) with a mean disease duration of 2.9 years (range 2‐5 years) were examined with [^11^C]*(R)*‐PK11195 PET and compared with 10 healthy controls.

**Results:**

Patients with the parkinsonian phenotype of MSA showed a significant (*P* ≤ 0.01) mean increase in binding potentials compared with healthy controls in the caudate nucleus, putamen, pallidum, precentral gyrus, orbitofrontal cortex, presubgenual anterior cingulate cortex, and the superior parietal gyrus. No correlations between binding potentials and clinical parameters were found.

**Conclusions:**

In early clinical stages of the parkinsonian phenotype of MSA, there is widespread microglial activation as a marker of neuroinflammatory changes without correlation to clinical parameters in our patient population. © 2019 The Authors. *Movement Disorders* published by Wiley Periodicals, Inc. on behalf of International Parkinson and Movement Disorder Society.

Multiple system atrophy (MSA) is a sporadically occurring, adult‐onset progressive neurodegenerative disease and presents clinically with variable combinations of marked autonomic insufficiency combined with a progressive akinetic‐rigid syndrome and/or cerebellar dysfunction.^1^, ^2^ The parkinsonian phenotype of MSA (MSA‐P) exhibits predominant parkinsonism, whereas MSA‐C has predominant cerebellar signs.

Histopathological findings in MSA show neuronal loss mainly in the nigrostriatal and olivopontocerebellar pathways, with α‐synuclein positive glial cytoplasmic inclusions^3^ associated with reactive astrocytes and activated microglia.^4^, ^5^, ^6^ α‐Synuclein aggregates are released from degenerating neurons, inducing neuroinflammation in the form of glial activation that results in cell loss and the formation of further aggregates and thus potentiates its neurodegenerating effect.^5^ Although the exact role of activated microglia in MSA and other neurodegenerative diseases is still a matter of discussion, there is evidence that microglia, once activated, continue to promote disease progression. Neuropathological findings demonstrate that microglial activation parallels system degeneration in MSA.^7^

Microglia are the resident immunocompetent cells of the CNS.^8^ They respond to a variety of pathological stimuli by developing phagic properties and express immunologically relevant molecules.^9^ As these cells react before signs of tissue damage are apparent, they provide an early marker of active disease. One of the molecules selectively expressed by activated microglia is the mitochondrial translocator protein 18‐kDa TSPO.^10^ In lesions with an intact blood‐brain barrier, activated microglia are the primary source of TSPO expression. (*R*)‐PK11195 (1‐[2‐chlorophenyl]‐N‐methyl‐N‐[1‐methylpropyl]‐3‐isoquinoline carboxamide) is a selective ligand for TSPO and, when labeled with [^11^C], can be used as a PET tracer.^11^

In a pilot study in patients with MSA using [^11^C]*(R)*‐PK11195 PET, we previously demonstrated increased microglial activation in the dorsolateral prefrontal cortex, putamen, pallidum, substantia nigra (SN) and pons in 5 patients with MSA.^12^

The aim of our present study was to assess the quantity and distribution of microglial activation in a large MSA patient cohort using a high‐resolution, high‐sensitivity PET camera and to correlate the extent of microglial activation with clinical parameters.

## Methods

Fourteen patients (7 female, 7 male; mean age, 58 years; range 45‐73 years) with MSA‐P according to consensus criteria^1^ were recruited for [^11^C](*R*)‐PK11195 PET. Mean disease duration (from the onset of first motor symptoms) was 2.9 years (range 2 to 5 years). For clinical details, see Table 1. Ten healthy controls (HCs; 4 female, 6 male, mean age, 59 years; range 38‐70 years) also had [^11^C](*R*)‐PK11195 PET. None of the HCs reported any relevant medical history, and all HCs had a normal neurologic examination.

**Table 1:** Clinical and demographic data of the MSA‐P cohort

Patient number	Age	Disease duration	UPDRS III	H + Y	S + E	Autonomic	Cerebellar	Pyramidal	l‐Dopa response	MSA‐P
1	45	2	28	2	100	OH	None	yes	No	Possible
2	54	5	52	4	40	OH, urinary urgency	None	None	Only initially	Probable
3	51	4	31	3	80	urinary frequency and urgency	None	None	Partial	Possible
4	53	2	28	2,5	90	Urinary incontinence, ED	None	None	No	Probable
5	64	4	44	3	70	Urinary incontinence	None	Yes	Partial	Possible
6	59	2	42	4	50	OH, urinary incontinence	None	None	Poor	Probable
7	59	3	41	3	70	Marginal OH	Slight action tremor	Yes	Poor	Possible
8	73	4	31	3	40	OH	None	Yes	Only initially	Probable
9	69	2.5	13	2,5	80	ED, urinary urgency, OH	None	None	Partial	Probable
10	47	2	14	2	80	ED, OH	None	None	No	Probable
11	57	2.5	31	2,5	70	Urinary frequency and urgency, OH	None	None	No	Probable
12	47	3	45	4	40	Urinary incontinence, OH	None	None	Poor	Probable
13	63	2	30	2,5	70	ED, urinary incontinence, OH	None	Yes	Poor	Probable
14	69	3	30	2,5	80	ED	None	None	No	Possible

ED, erectile dysfunction.

Overview of clinical patient data including age and disease duration at study participation in years, UPDRS III, Hoehn and Yahr stage (H + Y), and Schwab and England scale (S + E). Orthostatic hypotension (OH) is defined by a reduction of systolic blood pressure by at least 30 mm Hg or of diastolic blood pressure by at least 15 mm Hg within 3 minutes of standing from the recumbent position.

Ethical approval and permission were given by the Hammersmith Hospitals Trust Ethics Committee and the Administration of Radioactive Substances Advisory Committee of the UK Department of Health. Informed written consent was obtained from all study participants prior to enrollment. All procedures were conducted according to the Declaration of Helsinki.

### MRI

Volumetric T_1_‐weighted MR images were acquired for coregistration with PET to define anatomical regions of interest using a 1.0 T Picker HPQ scanner (Picker, Cleveland, OH).

### [^11^C](*R*)‐PK11195 PET

Participants underwent 3‐dimensional [^11^C](*R*)‐PK11195 PET using an ECAT EXACT HR++ (CTI/Siemens 966) camera located in the Cyclotron Building at Hammersmith Hospital, London, UK, with an axial field of view of 23.4 cm. The tomograph has a spatial resolution of 4.8 ± 0.2 mm FWHM (transaxial, 1 cm off axis) and 5.6 ± 0.5 mm (axial, on axis) after image reconstruction.^13^ Scanning procedures were performed according to previously established protocols (please refer to reference 14 and online supplement). Binding potential (BP), a measure of specific binding of the tracer (B_max_/K_d_ — available concentration of binding sites/receptor dissociation constant), was calculated at a voxel level using a simplified reference tissue model.^15^ For details, see reference 16 and online supplement.

### Image Analysis

**Figure 1:**
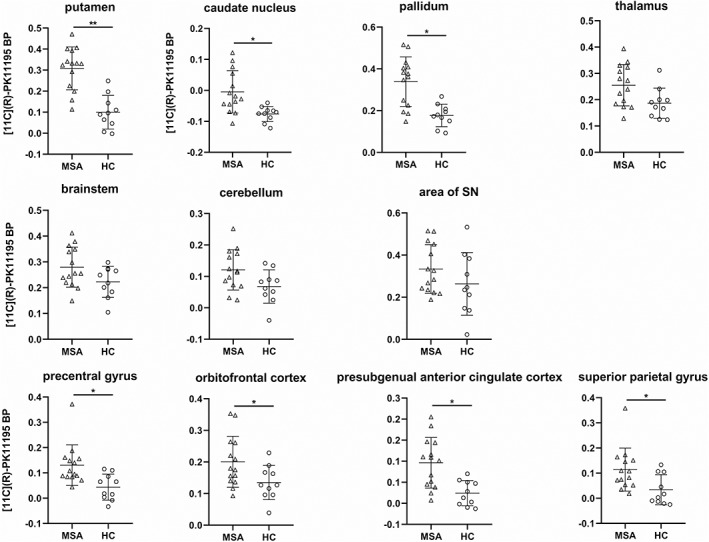
Comparison of binding potential (BP) values of MSA‐P patients and HCs. [^11^C]*(R)*‐PK11195 BP of MSA‐P patients in the 7 selected VOIs and selected cortical VOIs (mean of left and right, if given). Horizontal bars indicate mean and standard deviation. *P < 0.01, **P ≤ 0.001.

Parametric BP images were coregistered to the patients’ MRIs (see Fig. 1 in the online supplement). Volumes of interest (VOIs) were outlined on the MRI using a probabilistic atlas^17^ and applied to the corresponding BP maps using Analyze software.^18^ We defined the SN on MRI slices where clearly visible, but as its volume is small compared with PET resolution, anatomical definition was imprecise when coregistering the BP map to the MRI. To account for this inaccuracy, we use the term *area of SN*.

Mann‐Whitney *U* tests performed with SPSS 23 (SPSS Inc., Chicago, IL) were used to interrogate the presence of significant differences in mean BP values between groups, and a *Z* value was calculated for each VOI. Seven VOIs that were reported to be affected in MSA by previous studies (brain stem [includes the pons and medulla oblongata], cerebellum, caudate nucleus, putamen, pallidum, thalamus, and area of SN) were selected. Results were corrected for multiple comparisons using p‐plot on an α level of 0.05 (Hochberg correction).^19^

### Data Availability

The data sets generated and/or analyzed during the current study are available from the corresponding author on reasonable request.

## Results

### [^11^C]*(R)*‐PK11195 PET

A significant increase in microglial activation in MSA‐P patients compared with HCs (Fig. 1) was seen in the putamen (*P* = 0.001), caudate nucleus (*P* = 0.002), pallidum (*P* = 0.002), precentral gyrus (*P* = 0.004), orbitofrontal cortex (*P* = 0.006), presubgenual anterior cingulate cortex (*P* = 0.006), and superior parietal gyrus (*P* = 0.007). The other VOIs did not show a significant difference between MSA‐P patients and HCs. Comparison of BP between groups looking at the whole brain (*P* = 0.013), the parietal lobe (*P* = 0.016), and the frontal lobe (*P* = 0.022) revealed a trend toward an increase in BP in MSA‐P patients but did not reach statistical significance (p‐plot, α = 0.05).

### Correlation BP Clinical Parameters

Spearman correlation between BP in the selected VOIs and the clinical parameters age and disease duration at study participation, sum of the motor part (III) of the UPDRS, Hoehn and Yahr stage, and Schwab and England scale did not show significant results (*P* ≤ 0.05 was considered significant).

## Discussion

Neuroinflammation is a crucial part of the neuropathological changes in MSA,^7^ and [^11^C]*(R)*‐PK11195 PET allows detection of the location and extent of activated microglia in vivo. In our study, [^11^C]*(R)*‐PK11195 PET in patients with MSA‐P detected increased signals in regions known to be targeted by the disease: the striatum and pallidum. In addition, frontal and parietal regions also displayed increased BP. Interestingly, there was a trend toward an increase in BP on a whole‐brain level and in the frontal and parietal lobes. The failure to find a significant BP increase in the SN is in contrast to our earlier findings.^15^ However, 12 of our 14 patients showed an increase in this region compared with the mean BP value of HCs in at least 1 SN. Also, the patients in the previous study had longer mean disease duration than in our present study (68 versus 35 months) and were clinically more severely affected.

Although the changes in BP values in the cerebellums of MSA patients were not statistically significant when compared with those in the HCs, a number of patients showed raised values. This is of particular interest, as all our patients were classified as MSA‐P and only 1 of them had a slight tremor. Potentially, the raises in [^11^C]*(R)‐*PK11195 binding indicate a “preclinical” change. Studies in other neurological diseases have shown that microglial activation can precede clinical manifestations. Asymptomatic gene carriers have shown increased striatal and cortical BP in Huntington's disease.^20^ Patients with idiopathic rapid eye movement sleep behavior disorder showed an increase in BP in the left SN along with a decrease in f‐dopa uptake in the bilateral putamen compared with HCs.^21^ As most cases are converting to α‐synucleinopathies, these findings support the role of microglia as an early component of neurodegenerative disorders that could be a potential therapeutic target.

Our patients were clinically at an early stage of the disease, and we did not find a correlation between regional BP values and clinical severity. Most had a diagnosis of probable MSA‐P but showed some clinical heterogeneity. There was high intersubject variability for regional BP values, a phenomenon that has been observed in other neurodegenerative disorders that have been examined with TSPO PET tracers. The high variability of microglial activation is also reflected in PET studies of idiopathic Parkinson's disease, with some authors finding a correlation with clinical phenotype, but others failing to do so.^22^ Because of the lack of TSPO studies in MSA, we can only assume at this stage that the clinical phenotype and severity will not be a simple linear function of the amount and localization of microglial activation. Overall, microglial activation appears to happen early in neurological diseases, and the mutual relationship between neurodegeneration and neuroinflammation is complex. Ishizawa and colleagues suggest a distinction in the course of microglial activation between gray and white matter. They hypothesize that the amount of microglial activation in MSA is reduced with increasing severity of tissue injury in white‐ but not in gray‐matter areas, where such correlations could not be shown.^6^ Also, although some authors assume that microglial activation is part of pathogenesis, it might also be that these cells play a protective role initially, as has been postulated for patients with early‐stage Alzheimer's disease.^23^, ^24^

To further elucidate the complex interaction between microglial activation and clinical phenotype and progression in MSA and other neurodegenerative disorders, longitudinal combined clinical and imaging studies are needed that also investigate other aspects of microglial activation apart from TSPO expression.^25^

When interpreting our results, it is important to realize that TSPO expression is only one part of the much more complex phenomenon of microglial activation. We currently do not have in vivo imaging methods available that allow distinguishing between different types of activated micro‐ and astroglia. In our study, we used [^11^C]*(R)*‐PK11195 as a marker of TSPO rather than one of the second‐generation TSPO PET tracers. [^11^C]*(R)*‐PK11195 is the best characterized TSPO tracer and has enabled us to compare our results with those of previous studies of microglial activation in α‐synucleinopathies. Also, its binding affinity is not influenced by the TSPO polymorphism. However, [^11^C]*(R)*‐PK11195 has a lower specific‐to‐background signal ratio than newer microglial tracers, which might lead to underestimation of the extent of microglial activation.^26^

We provide evidence that microglial activation is an important feature of the pathological changes in MSA. Decoding the role of neuroinflammation in the pathophysiology of neurodegenerative diseases is a crucial step toward possible therapeutic strategies.

## Author contributions

1. Research project: A. Conception, B. Organization, C. Execution

2. Statistical Analysis: A. Design, B. Execution, C. Review and Critique

3. Manuscript Preparation: A. Writing of the first draft, B. Review and Critique

D. Kübler: 2A, 2B, 3A.

T. Wächter, N. Cabanel, Z. Su: 1B, 1C, 2B.

F. Turkheimer: 2B, 2C.

R. Dodel, D.J. Brooks, W.H. Oertel: 1A, 3B.

A. Gerhard: 1A, 1B, 1C, 2A, 3B.

## Financial Disclosures of all authors (for the preceding 12 months)

Dorothee Kübler has received grants from BIH‐Charité Clinician Scientist Program and is employed by Charité‐Universitätsmedizin Berlin. Tobias Wächter has received honoraria from UCB and BIA and is employed by Passauer Wolf Bad Gögging. Nicole Cabanel is employed by Vitos Clinic for Psychiatry and Psychotherapy Giessen, Germany. Zhangjie Su is employed by University College London Hospitals NHS Foundation Trust. Federico E. Turkheimer has received grants from Wellcome NIMA consortium, BBSRC project grant ERC ‐IMI2, MRC project grant, and ARSEP project grant. Richard Dodel has received honoraria from Pfizer and Studienstiftung des deutschen Volkes, grants from Bundesministerium für Bildung und Forschung and Deutsches Zentrum für neurodegenerative Erkrankungen, Landscape, Clasp, and the Michael J. Fox Foundation, and different issued patents for the diagnosis and treatment of Alzheimer's and other neurodementing diseases. David J. Brooks owns stock in GE Healthcare, is a consultant for GE Healthcare and Biogen, is on the advisory boards of Alzheimer UK, Alzheimer Research UK, and Biogen, has received honoraria from Biogen and Zambon, has received grants from Danish Council, Lundbeck, Parkinson Denmark, GE, and Parkinson UK, and is employed by Newcastle University and Aarhus University. Wolfgang H. Oertel owns stock in BiogenIdec, Lilly, Medigene, Merck Darmstadt, Morphosys, and Roche, is a consultant for Adamas and Novartis, is on advisory boards of Adamas, BristolMyerSquibb, Eisai, GEHealth, Mundipharma, Novartis, Roche, and UCB, has received honoraria from AbbVie, Desitin, Mundipharma, Novartis, Prothena, and UCB, has received grants from Charitable Hertie Foundation, the German Ministry of Education and Research, the German Research Foundation, the Michael J Fox Foundation, International ParkinsonFonds, ParkinsonFonds Deutschland, and Novartis Pharma Germany, and is employed by Philipps‐University Marburg. Alexander Gerhard has received grants for P2X7 receptor‐based microglia PET imaging in Parkinson's Disease (PRI‐PD), the Michael J. Fox Foundation; the UK Genetic Frontotemporal Dementia Initiative (UK GENFI), MRC; Imaging of Neuroinflammation in Neurodegenerative Diseases (INMiND), European Commission FP7; and imaging cerebral neuroinflammation in acute and chronic cerebrovascular disease, MRC/NIHR; and is employed by Elisabeth Krankenhaus, Essen, Germany; University Hospital Essen, Germany; and University of Manchester, Manchester, UK.

## Supporting information


**Supplementary Figure 1** Example of BP in MSA
