# Supplementary material for: Widespread microglial activation in multiple system atrophy
Source: Mov Disord. 2019 Feb 6;34(4):564–8. doi: 10.1002/mds.27620 (PMC6659386; doi:10.1002/mds.27620)
Supplement: Supplementary file 1 — Supplementary Figure 1 Example of BP in MSA [file MDS-34-564-s001.docx]

**[^11^C](*R*)-PK11195 PET – data acquisition and processing:**

A transmission scan, used to correct for attenuation of emitted radiation by skull and soft tissues, was acquired using a single rotating photon point source of 150 MBq of ^137^Cs. 30 seconds after, 282.5 MBq ± 30.1MBq of [^11^C](*R*)-PK11195 in 5ml normal saline were infused intravenously over 10 seconds. Mean molar activity was 84.8GBq/µmol. Three-dimensional sinograms of emission data were acquired over 60 minutes as 18 time frames. Patients were placed in the scanner orientated parallel to the orbitomeatal line and head positioning was monitored throughout the scan.

To identify the expected widespread distribution of pathological changes in MSA-P, cluster analysis was used to extract and identify a normal brain grey matter reference input function for each individual case as previously described ^12,16^. The patient cluster providing their “normal” cortical reference time activity curve (TAC) was tested for dissimilarity with a previously established normal mean population input TAC (*X*^2^ test, p<0.05); for details see ^16^.

**Supplementary Figure 1: Example of BP in MSA**


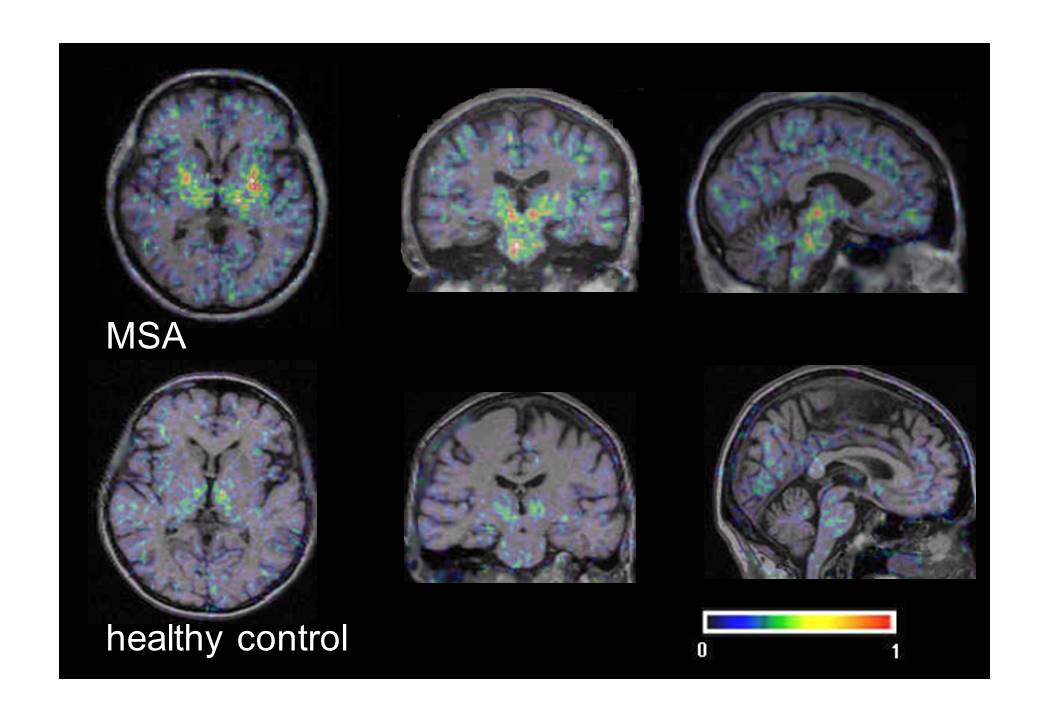


[^11^C]*(R)*-PK11195 binding potential (BP) values in a patient with MSA (top row) in comparison to a healthy control (bottom row) in transverse, coronal and sagittal section. Binding potential maps are registered with the individual MRI. Increased binding can be seen in basal ganglia, brainstem and cortical areas. The colour scale denotes BP values from 0 to 1.
